# Novel Arabidopsis microtubule-associated proteins track growing microtubule plus ends

**DOI:** 10.1186/s12870-017-0987-5

**Published:** 2017-02-02

**Authors:** Jeh Haur Wong, Takashi Hashimoto

**Affiliations:** Graduate School of Biological Sciences, Nara Institute of Science and Technology, Takayama 8916-5, Ikoma, Nara, 630-0192 Japan

**Keywords:** Arabidopsis, EB1, GPT1, GPT2, Plus-end tracking proteins, SPIRAL1

## Abstract

**Background:**

Microtubules (MTs) are polarized polymers with highly dynamic plus ends that stochastically switch between growth and shrinkage phases. In eukaryotic cells, a plethora of MT-associated proteins (MAPs) regulate the dynamics and higher-order organization of MTs to mediate distinct cellular functions. Plus-end tracking proteins (+TIPs) are a group of MAPs that specifically accumulate at the growing MT plus ends, where they modulate the behavior of the MT plus ends and mediate interactions with cellular targets. Although several functionally important + TIP proteins have been characterized in yeast and animals, little is known about this group of proteins in plants.

**Results:**

We report here that two homologous MAPs from *Arabidopsis thaliana*, Growing Plus-end Tracking 1 (GPT1) and GPT2 (henceforth GPT1/2), contain basic MT-binding regions at their central and C-terminal regions, and bind directly to MTs in vitro. Interestingly, GPT1/2 preferentially accumulated at the growing plus ends of cortical MTs in interphase Arabidopsis cells. When the GPT1/12-decorated growing plus ends switched to rapid depolymerization, GPT1/2 dissociated from the MT plus ends. Conversely, when the depolymerizing ends were rescued and started to polymerize again, GPT1/2 were immediately recruited to the growing MT tips. This tip tracking behavior of GPT proteins does not depend on the two established plant + TIPs, End-Binding protein 1 (EB1) and SPIRAL1 (SPR1).

**Conclusions:**

The Arabidopsis MAPs GPT1 and GPT2 bind MTs directly through their basic regions. These MAPs track the plus ends of growing MTs independently of EB1 and SPR1 and represent a novel plant-specific + TIP family.

**Electronic supplementary material:**

The online version of this article (doi:10.1186/s12870-017-0987-5) contains supplementary material, which is available to authorized users.

## Background

Microtubules (MTs) are highly conserved polarized cytoskeletal polymers that mediate cellular processes such as motility, cell division, polarity, intracellular transport, and signaling in eukaryotic cells. GTP-bound α/β-tubulin heterodimers are stacked in a head-to-tail fashion to form polar protofilaments, which associate laterally to form polar hollow MT cylinders with highly dynamic plus ends and more stable minus ends. The GTP bound to β-tubulin is hydrolyzed to GDP as MT assembly progresses. Before hydrolysis takes place, the GTP-bound tubulin forms a cap that stabilizes the plus end of the MT and thereby promotes its growth. Stochastic loss of the stabilizing cap renders the MT lattice of GDP-bound tubulin unstable and causes rapid MT depolymerization, a process known as catastrophe. When GTP-tubulin subunits reassemble at the depolymerizing plus end, the MT resumes growth, a process known as rescue. This MT behavior of alternating between catastrophe and rescue at the plus end is known as dynamic instability [1, 2].

GTP-tubulin dimers adopt a curved conformation in solution and are thought to assemble into outwardly curved sheets at the extreme plus end [3]. As the number of laterally associating protofilaments increases, the curved GTP-tubulin sheets gradually straighten, to form a tubular structure. GTP hydrolysis and subsequent phosphate release occur soon after tubulin incorporation into the protofilament, resulting in a short GTP cap [4].

A subgroup of MT-associated proteins (MAPs) is characterized by their ability to accumulate at MT ends [5, 6]. Plus-end tracking proteins (+TIPs) recognize and bind to the plus-ends of MTs, and potentially modulate MT assembly dynamics and interactions between the MT plus ends and subcellular targets. Two categories of + TIPs can be distinguished; whereas “autonomous tip trackers” bind MT ends independently of other MAPs, “hitchhikers” are targeted to the MT ends through binding to an autonomous tip tracker, and they often also have a considerable binding affinity for the MT lattice. End-binding proteins (EBs) are autonomous tip trackers that regulate + TIP networks in animal cells by recruiting various hitchhiking + TIPs to the growing plus ends [7]. EBs bind in close proximity to the exchangeable GTP-binding site of tubulin, and are thought to sense the MT’s nucleotide state (GTP or GDP) [8, 9]. EB1 is probably associated with the GTP cap and GTP-like-tubulin, in which hydrolyzed GTP remains in an intermediate GDP-Pi state [10]. EBs show a characteristic localization pattern at the growing MT ends; their abundance peaks at the MT tip regions and gradually declines toward the main body of MTs, forming a “comet”-like pattern.

Plants have two types of EBs that localize either to the cytoplasm or nucleus. In *Arabidopsis thaliana*, cytoplasmic EB1a and EB1b and nuclear EB1c all accumulate at the growing plus ends of MTs [11–15]. Knockdown or knockout of *EB1c* compromised the alignment of spindle and phragmoplast MTs in Arabidopsis root cells, and caused frequent lagging of separating chromosomes at anaphase in cultured *Nicotiana tabacum* (tobacco) cells [15]. However, Arabidopsis mutants of *EB1a* and *EB1b*, as well as their double mutants, are almost indistinguishable from wild-type plants with respect to growth and morphology, indicating that EBs may not be important for MT functions in interphase plant cells [14, 15].

In addition to EB1, several other plant MAPs show partial accumulation at the MT plus ends [16]. SPIRAL1 (SPR1) is a plant-specific protein that belongs to a six-member family with overlapping functions in Arabidopsis [17–19]. SPR1 is localized to the MT lattice and also accumulates at the growing plus ends of MTs in Arabidopsis cells, forming an extended comet-like pattern that is much longer than that formed by EB1 [18]. The in vivo association of SPR1 with cortical MTs does not require EB1 [20]. It is not clear how SPR1 associates with MTs and how it is preferentially recruited to the plus ends, because SPR1 is not recovered in MAP preparations from Arabidopsis cell extracts [21] and recombinant SPR1 protein does not show MT binding in vitro [16]. The Arabidopsis *spr1* mutant shows right-handed helical growth of elongating axial organs [22], and this helical phenotype is exaggerated in multiple mutants of the *SPR1* gene family [19].

We previously isolated several novel MAPs from Arabidopsis cultured cells [21]. Among them, a putative MAP encoded by At3g53320 was found to localize to cortical MTs when transiently expressed as a GFP fusion in *Allium cepa* (onion) epidermal cells [21]. In Arabidopsis, there is another homologue (encoded by At2g37070) of this putative MAP. We named this homologue GPT1 (for Growing Plus-end Tracking protein 1) and the formerly identified MAP [21] GPT2. The amino acid sequences of GPT1 and GPT2 (henceforth GPT1/2) share 59.8% similarity and 46.1% identity, and do not contain any defined domains with known functions. In this report, we demonstrate that these GPT proteins are novel plant-specific + TIPs that track growing MT plus ends independently of EB1 and SPR1.

## Results

### MT-binding regions in GPT1/2

**Fig. 1:**
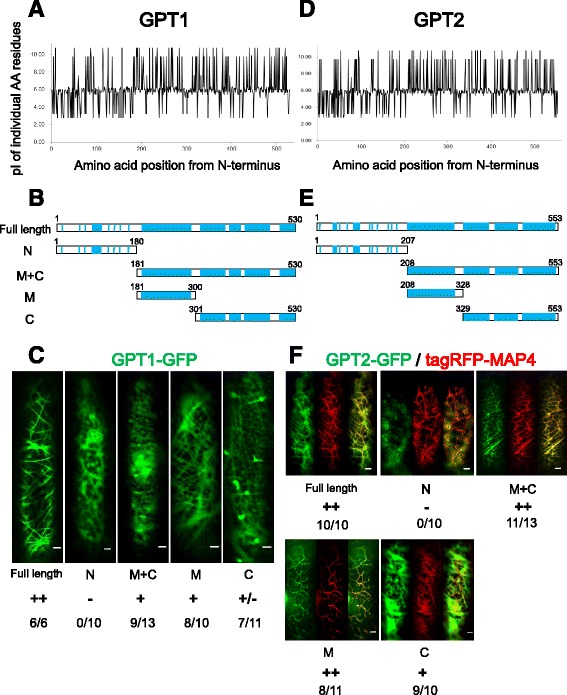
The positively charged domains of GPT1/2 bind MTs in onion epidermal cells. Full-length and truncated fragments of GPT1/2 were fused to GFP at their C-termini, and transiently expressed in onion epidermal cells. **a** and **d** Charge plots of GPT1 (**a**) and GPT2 (**d**). **b** and **e** Protein structures of GPT1 (**b**) and GPT2 (**e**). The positions of basic amino acid residues are indicated by blue lines. The N-terminal (N) regions are negatively charged, whereas the middle (M) and C-terminal (C) regions are positively charged. **c** and **f** Localization of GFP-fused GPT fragments to cortical MTs is indicated by ++ (strong localization), + (substantial localization), +/- (weak localization), and – (no localization). The numerator and denominator show the number of cells with positive localization patterns and the number of total cells that expressed GFP fusion proteins, respectively. The GPT2-GFP fusion (**f**) was co-expressed with tagRFP-MAP4 to visualize MTs. Left panels, GPT2-GFP; middle panels, tagRFP-MAP4; right panels, merged images. Scale bars, 10 μm

Although GPT1/2 do not exhibit any significant amino acid homology with functionally characterized proteins [21], we noticed that the middle (M) and C-terminal (C) regions of these proteins are enriched in the basic amino acid residues Arg, Lys, and His (18.9% in GPT1 and 17.9% in GPT2; Fig. 1a and b). By contrast, the N-terminal (N) regions are abundant in acidic Asp and Glu (22.2% in both GPT1/2). Thus, GPTs are polar proteins with short, negatively charged regions at their N-termini (pI values of 4.4 for GPT1 and 4.5 for GPT2) and longer, positively charged regions in their middles and C-termini (pI values of 10.7 for GPT1 and 10.6 for GPT2).

To identify MT-binding regions in these proteins, we fused full-length and truncated versions of GPT1 and GPT2 to GFP, and transiently expressed these fusions in onion epidermal cells, together with the red fluorescent MT marker tagRFP-MAP4 (Fig. 1). Dual color visualization of GPT1-GFP and tagRFP-MAP4 proved difficult, possibly due to GPT1 having a weak MT-binding capacity, competition between GPT1 and MAP4 for overlapping MT binding sites, or both. Therefore, the MT-binding capacity of GPT1-GFP was determined based on the presence of fine filaments (presumably representing cortical MTs decorated by GPT1-GFP). Colocalization of the GFP and RFP signals confirmed that full-length GPT2 localized to cortical MTs (Fig. 1f). The N-terminal (N) regions of GPT1 (1–180) and GPT2 (1–207) did not localize to MTs, whereas the N-terminal-deleted (M + C) (181–530 for GPT1, and 208–553 for GPT2) and the middle (M) (181–300 for GPT1, and 208–328 for GPT2) fragments were clearly localized to cortical MTs (Fig. 1f). The C-terminal (C) fragments (301–530 for GPT1, and 329–553 for GPT2) were somewhat associated with MTs, but to a lesser extent than the fragments containing the M region. These results from transient expression assays indicate that the positively charged basic amino acid residues (especially in the M region) mediate the binding of GPT1/2 to MTs in vivo.

### MT co-sedimentation assay

**Fig. 2:**
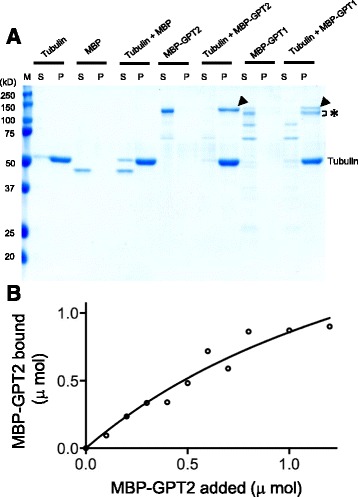
Recombinant GPT1 and GPT2 bind to MTs in vitro. (**a**) Purified MBP-fused GPT1 and GPT2 proteins were incubated with or without taxol-stabilized MTs, pelleted by ultracentrifugation, and analyzed by SDS-PAGE and Coomassie Brilliant Blue staining. The positions of full-length MBP-GPT1 and MBP-GPT2 are indicated by arrowheads, and the MT-binding partial degradation products of MBP-GPT1 are indicated with an asterisk. Tubulin is also indicated. S, supernatant fraction; P, pellet fraction; and M, size markers. (**b**) Quantitative analysis of the binding of MBP-GPT2 to MTs. Various concentrations of purified MBP-GPT2 was mixed with taxol-stabilized MTs and then subjected to co-sedimentation analysis, as in A. Assuming that there is one GPT2 binding site on the tubulin dimer, the binding equation (see Methods) was fitted

To test whether GPT1/2 directly bind to MTs, we fused full-length GPT1 and GPT2 to maltose-binding protein (MBP), expressed the recombinant proteins in bacteria, and purified the proteins by affinity purification using amylose resins. Some degradation occurred during the purification, particularly in the case of MBP-GPT1. When 1 μM of purified proteins was incubated with MTs assembled from bovine brain tubulin and pelleted by ultracentrifugation, intact MBP-GPT1 and MBP-GPT2 were identified in the microtubule pellet fraction (P) (arrowheads in Fig. 2a). The partial degradation products of MBP-GPT1 (asterisk in Fig. 2a) were also pelleted, whereas further degraded MBP-GPT1 and MBP-GPT2 fragments were not. In the absence of MTs, MBP-GPT1 and MBP-GPT2 remained in the supernatant (S). MBP alone was also evaluated to verify that it is not responsible for binding of GPT1/2 to MTs. As shown in Fig. 2, MBP remained in the supernatant fraction after ultracentrifugation. These results show that GPT1/2 bind MTs directly.

To determine the stoichiometry and affinity of GPT toward MTs, various amounts of MBP-GPT2 were centrifuged with a constant amount of MTs that were polymerized from 1 μM tubulin heterodimer, and a binding curve was obtained (Fig. 2b). MBP-GPT2 bound to MTs in a concentration-dependent manner and saturated at a stoichiometry of approximately 2.5 +/− 0.9 mol of GPT2 per mol of tubulin dimer. The dissociation constant K_d_ was calculated to be 1.9 +/− 1.0 μM. However, if GPT2 associates with MTs in more than one binding mode with different affinities (as indicated below), this simple regression analysis does not provide true binding values.

### Subcellular localization

To determine the subcellular localization of GTP1/2 and to monitor their dynamics in vivo, GFP was fused to either the N-terminus or the C-terminus of GPT1 and expressed under the constitutive cauliflower mosaic virus 35S promoter, because the 5′-regulatory region of *GPT1* was recalcitrant to cloning, whereas GFP was fused to the C-terminus of GPT2 and expressed under its native promoter. Both N-terminal and C-terminal GFP fusions of GPT1 localized to MTs in transient expression assays using onion epidermal cells (Additional file 1), indicating that the location of GFP does not affect the subcellular localization of fusion proteins. The Arabidopsis MT marker line that expresses mCherry fluorescent protein fused to β-tubulin 6 (mCherry-TUB6) was used as a transformation host.

**Fig. 3:**
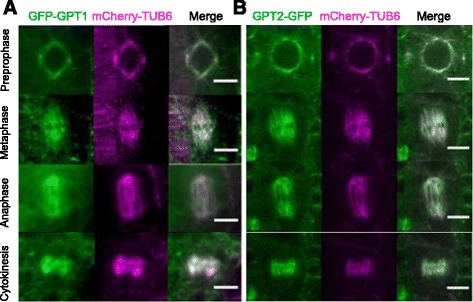
GPT1/2 decorate mitotic MT structures in Arabidopsis root epidermal cells. GFP fusions (*green*) were expressed in transgenic Arabidopsis plants harboring the mCherry-TUB6 MT marker (*magenta*). Epidermal cells in the cell division zone of the roots of seedlings were analyzed. Dividing cells at preprophase, metaphase, anaphase, and cytokinesis are shown. **a** GFP-GPT1 was expressed under the control of the cauliflower mosaic virus 35S promoter. **b** GPT2-GFP was expressed under the control of the endogenous regulatory elements of *GPT2*. Scale bars, 5 μm

GFP-GPT1 and GTP2-GFP co-localized with mCherry-TUB6 and decorated the nuclear envelope and preprophase bands, mitotic spindles, and phragmoplasts of the mitotic cells of the root meristem (Fig. 3). GFP-GPT1 labeling differed slightly from mCherry-TUB6 labeling, particularly in expanding phragmoplasts. This observation prompted us to carefully examine the localization and dynamics of GPT1 and GPT2 in cortical MT arrays, where the dynamics of single MTs can be clearly visualized.

### GPT1 and GPT2 are novel + TIPs

**Fig. 4:**
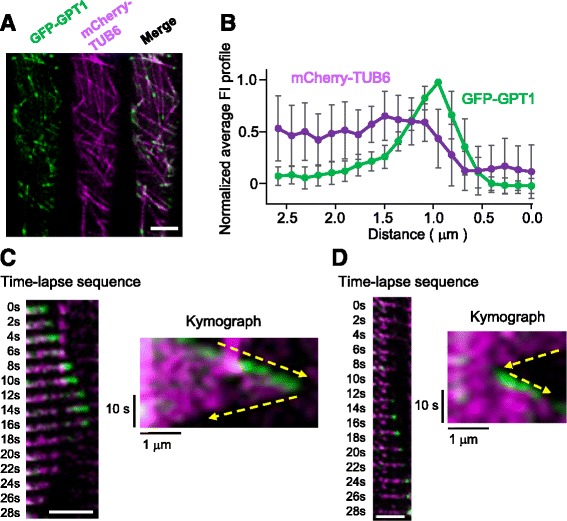
GPT1 tracks the growing plus ends of cortical MTs. **a** The subcellular localization of GFP-GPT1 (*green*) and mCherry-TUB6, which labels cortical MTs (*magenta*), was analyzed in interphase cells of the Arabidopsis root epidermis. Scale bar, 5 μm. **b** Average fluorescence intensity (FI) profiles of GFP-GPT1 and mCherry-TUB6 were obtained by analyzing and plotting data from 20 MT images. The data were normalized and the peak intensity of GFP-GPT1 was set to 1. The error bars indicate SEM. **c** Representative time-lapse sequence and corresponding kymograph of a MT that underwent catastrophe at t = 14 s. GFP-GPT1 disappeared from the tip immediately after the MT began to depolymerize. Dashed *yellow arrows* indicate the point at catastrophe occurred. Scale bar, 2.5 μm. **d** Representative time-lapse sequence and corresponding kymograph of a MT that underwent rescue at t = 12 s. GFP-GPT1 localized to the tip immediately after MT growth resumed. *Dashed yellow arrows* indicate the point at which rescue occurred. Scale bar, 2.5 μm



Time-lapse imaging of root epidermal cells using a spinning disk confocal microscope revealed that GFP-GPT1 and GPT2-GFP not only decorated the MT lattice, but also accumulated as particles on MTs (Fig. 4 and Additional files 2, 3 and 4). Double intensity plots with MTs (mCherry-TUB6) and GFP-labeled GPT1/2 showed that GPT1/2 are localized to the MT ends in a comet-like pattern, with the highest signal intensity at the MT ends and a gradual decline in signal further from the MT ends. This MT-end localization was more pronounced for GFP-GPT1 than for GPT2-GFP. The MT ends labeled with GFP were highly dynamic, indicating that GPT1/2 both label the plus ends of cortical MTs. GFP-GPT1 was associated with polymerizing plus ends, and dissociated as soon as catastrophe occurred (Fig. 4c). However, when the MTs started to polymerize again, GFP-GPT1 was immediately recruited to the growing ends (Fig. 4d). GPT2-GFP showed a similar but weaker labeling pattern when compared with GFP-GPT1 (Additional file 2). These results demonstrate that GPT1 and GPT2 preferentially recognize the plus ends of growing MTs.

**Additional file 3: Movie S1.** Dynamics of cortical MTs (magenta; mCherry-TUB6) and GFP-GPT1 (green) in epidermal cells of the roots of Arabidopsis seedlings. The image sequence corresponds to Fig. 4a. (AVI 14800 kb)




**Additional file 4: Movie S2.** Dynamics of cortical MTs (labelled magenta by mCherry-TUB6) and GPT2-GFP (green) in epidermal cells of the cotyledons of Arabidopsis seedlings. The image sequence corresponds to Additional file 2: Figure S2A. (AVI 11123 kb)


**Fig. 5:**
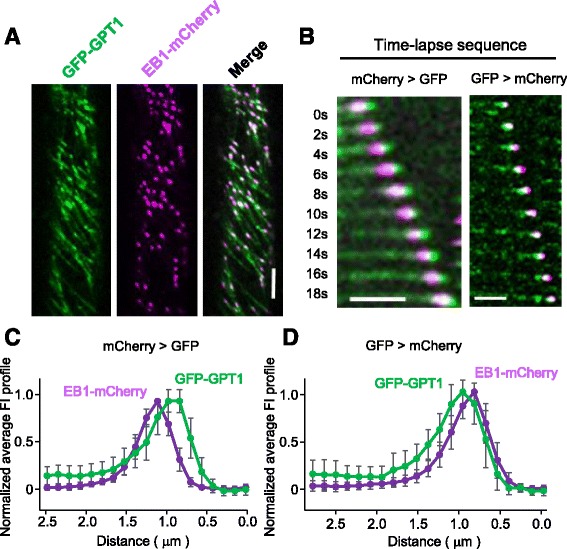
GPT1 and EB1 label similar regions of growing MT ends. **a** Subcellular localization of GFP-GPT1 (*green*) and EB1-mCherry (*magenta*) was analyzed in interphase cells of the Arabidopsis root epidermis. Scale bar, 5 μm. **b** Representative time-lapse sequences. *Left*, mCherry signals were analyzed first, followed by GFP signals. *Right*, GFP signals were analyzed first, followed by mCherry signals. Scale bars, 2.5 μm. **c** and **d** Average FI profiles of GFP-GPT1 and EB1-mCherry were obtained by analyzing and plotting data from 20 MT images. The data were normalized and the peak intensities of GFP-GPT1 and EB1-mCherry were set to 1. The error bars indicate SEM. **c** mCherry signals were analyzed first, followed by GFP signals. **d** GFP signals were analyzed first, followed by mCherry signals



EB1 is a + TIP family member that recognizes the GTP-cap region of growing MTs [7]. To compare the labeling patterns of GPT1/2 with that of EB1, we stably co-expressed GFP-labeled GPT1 or 2 and mCherry-labeled Arabidopsis EB1b (EB1-mCherry) in Arabidopsis plants. EB1-mCherry showed strong plus-end labeling, as previously reported [11–15]. Many, but not all, EB1 particles co-localized with GFP-GPT1 (Fig. 5a; Additional file 5). When the relative fluorescent intensities of GFP and mCherry were plotted, the comet-shaped fluorescent signals of GFP-GPT1 and EB1-mCherry partially overlapped. In our confocal microscopy setup, the two emitted fluorescent signals from GFP and mCherry were sequentially detected after alternately exciting each fluorophore. When mCherry fluorescence was detected for 0.2 s, followed by the detection of GFP signal for 0.5 s, the highest GFP-GPT1 signal intensity was located approximately 0.2 μm closer to the MT end when compared with the highest EB1-mCherry signal (Fig. 5b, c). When the detection order was reversed, however, the highest EB1-mCherry signal intensity was located approximately 0.15 μm in front of the highest signal intensity of GFP-GPT1 (Fig. 5b, d). At the current spatial resolution, optical artifacts associated with our detection system make it difficult to conclusively determine whether EB1 and GPT1 completely co-localize at growing MT plus ends. In both observations, the fluorescent signal intensities of the EB1 comets decreased gradually and reached background levels at 1.5 μm behind the tips. By contrast, substantial levels of GFP-GPT1 signal (approximately 20% of the highest intensities) remained associated with the MT lattice behind the comet tails.

**Additional file 5: Movie S3.** Dynamics of EB1-mCherry (magenta) and GFP-GPT1 (green) in epidermal cells of the roots of Arabidopsis seedlings. In one image acquisition, mCherry fluorescence was first recorded for 0.2 s and then GFP fluorescence was recorded for 0.5 s. The image sequence corresponds to Fig. 5a. (AVI 13786 kb)


### Tip-tracking of GPT does not require EB1 or SPR1

**Fig. 6:**
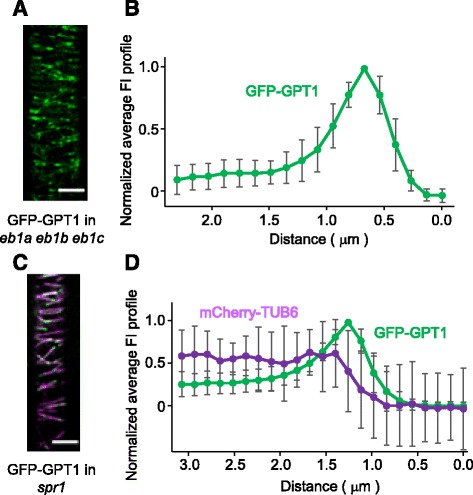
GPT1 does not require EB1 or SPR1 to track the MT end. **a** and **b** GFP-GPT1 was expressed in the Arabidopsis *eb1a eb1b eb1c* triple mutant. **a** The subcellular localization of GFP-GPT1 was analyzed in interphase cells of the Arabidopsis root epidermis. Scale bar, 5 μm. **b** An average FI profile of GFP-GPT1 was obtained by analyzing and plotting data from 20 MT images. The data were normalized and the peak intensity of GFP-GPT1 was set to 1. The error bars indicate SEM. **c** and **d** GFP-GPT1 was expressed in the *spr1* mutant that also expressed mCherry-TUB6 as a MT marker. **c** The subcellular localization of GFP-GPT1 and mCherry-TUB6 was analyzed in interphase cells of the Arabidopsis root epidermis. Scale bar, 5 μm. **d** Average FI profiles of GFP-GPT1 and mCherry-TUB6 were obtained by analyzing and plotting data from 20 MT images. The data were normalized and the peak intensity of GFP-GPT1 was set to 1. The error bars indicate SEM



EB1 recognizes a structural feature of the MT GTP cap and directly binds to the plus ends of growing MTs both in vitro and in vivo [7]. Many animal + TIPs do not bind directly to the MT plus ends in vivo, but are recruited to this region by EB1. To test whether GPT accumulates at the MT plus end directly or through a hitchhiking mechanism involving interaction with EB1, we analyzed the tip-tracking behavior of GPT in the Arabidopsis *eb1* null mutant. Because the Arabidopsis genome includes three *EB1* genes that may function redundantly [14], a triple *eb1* knockout mutant was used to study the expression of GFP-GPT1 and GPT2-GFP. GPT1/2 both localized to the plus ends of growing MTs, in the same pattern as observed in the wild-type background (Fig. 6a, b; Additional files 6, 7 and 8).

**Additional file 7: Movie S4.** Dynamics of GFP-GPT1 in root epidermal cells of the *eb1* triple mutant in Arabidopsis. The image sequence corresponds to Fig. 6a. (AVI 14176 kb)




**Additional file 8: Movie S5.** Dynamics of GPT2-GFP in cotyledon epidermal cells of the *eb1* triple mutant in Arabidopsis. The image sequence corresponds to Additional file 6: Figure S3A. (AVI 13792 kb)


SPR1 and its homologues are plant-specific MT-localized proteins that bind to both the growing plus ends and the MT lattice [17, 18]. Arabidopsis contains seven SPR1 homologues that may be redundant, and SPR1 contributes predominantly to the anisotropic growth of seedling roots [19]. Therefore, we used a *spr1* null mutant to test whether SPR1 is required for the plus-end tracking behavior of GPT. Cortical MTs were labeled with mCherry-TUB6. In the *spr1* mutant, GPT1/2 labeled growing MT plus ends in interphase epidermal cells, forming a comet-like pattern (Fig. 6c, d; Additional files 6, 9 and 10). The tip-tracking behavior of the GFP-labeled GPTs in the *spr1* null mutant was indistinguishable from that in wild-type epidermal cells.



**Additional file 9: Movie S6.** Dynamics of cortical MTs (labelled magenta by mCherry-TUB6) and GFP-GPT1 (green) in root epidermal cells of the *spr1* mutant in Arabidopsis. The image sequence corresponds to Fig. 6c. (AVI 8429 kb)




**Additional file 10: Movie S7.** Dynamics of cortical MTs (labelled magenta by mCherry-TUB6) and GPT2-GFP (green) in root epidermal cells of the *spr1* mutant in Arabidopsis. The image sequence corresponds to Additional file 6: Figure S3C. (AVI 14930 kb)


These results demonstrate that EB1 and SPR1 are not required for the MT plus-end tracking function of GPT1 and GPT2.

## Discussion

### GPT1/2 bind to MTs through its basic regions

With the exception of the N-terminal regions, GPT1/2 are relatively rich in Lys, Arg, and His residues, with GPT1/2 having average pI values of 10.7 and 10.6, respectively. These positively charged residues are distinct biochemical features of GPTs; however, these values are lower than those of the highly basic domains of some MAPs, such as BPP1, BPP2, and BPP3, which have pI values of 12.9–13.4 [21]. Transient expression of GFP-tagged full-length and truncated GPT1/2 proteins in onion epidermal cells showed that the basic regions of GPT1/2 are responsible for the in vivo localization to cortical MTs. The central and C-terminal basic regions were independently targeted to MTs in vivo, suggesting that several MT-binding regions exist in GPT1/2. Because recombinant GPT1/2 proteins bind to MTs in vitro, the basic regions appear to bind MTs directly.

Various MT-binding regions have been identified in MAPs, and a high pI value resulting from basic amino acid residues was found to be characteristic of the MT-binding regions of certain MAPs [16]. The positively charged regions are thought to interact electrostatically with the highly acidic Glu- and Asp-rich C-terminal tails of α- and β-tubulins. Because an acidic C-terminus is a common feature of all eukaryotic tubulins [23], this charge-based MT-binding mode is frequently used by MAPs in various organisms.

### GPT preferentially accumulates at the plus ends of growing MTs

GFP-tagged GPT not only labels the MT lattice, but is considerably enriched at the growing plus ends of cortical MTs. This plus-end localization is stronger for GFP-GPT1 than for GPT2-GFP. We do not know whether the affinity for the plus end is inherently different between GPT1/2, or whether the location of the fused GFP molecule influenced the biochemical properties of GPT1/2. Although GPT1/2 have highly similar distributions of basic amino acids and considerable levels of amino acid sequence identity, we noticed that GPT1-GFP associates less efficiently with MTs than does GPT2-GFP when a MT marker is co-expressed in onion epidermal cells. Further biochemical analyses are required to identify possible differences between MT plus-end targeting by GPT1/2.

The tip-tracking behavior of GPT1/2 and the canonical + TIP, Arabidopsis EB1, was compared by coexpressing these proteins tagged with different fluorescent markers in Arabidopsis plants. EB1 specifically recognized the growing MT plus end and dissociated almost completely at 1.5 μm from the MT tip, where there are no binding sites for EB1 [8–10]. By contrast, the GPT1/2 tip-tracking fluorescent signal decreased with increasing distance from the MT plus end, but a substantial proportion of GPT1/2 molecules remained bound to the GDP-tubulin MT lattice. This propensity of GPT1/2 to bind to the MT lattice may obstruct observations of tip-localized GPT1/2 in dense mitotic MT arrays. Although further analysis at higher resolution is necessary, our present localization data suggest that EB1 and GPT1 have distinct binding sites at growing MT plus ends. EB1 recognizes the terminal GTP-cap and an extended region of several hundreds of tubulin molecules at growing MT ends [8, 9]. Recent imaging of EB1 localization using cryo-electron tomography showed that EB1 interacts with outwardly curved and straight regions of the MT lattice that probably represent GTP- and GDP-Pi-tubulin dimers, respectively, at the MT ends [10]. EBs bind at the intersection of four tubulin dimers in two adjacent protofilaments [8, 9]. This binding site is ideally suited for sensing the nucleotide states of the surrounding tubulin dimers. To autonomously track the growing MT ends, a + TIP should recognize subtle structural changes of tubulins that are associated with the nucleotide status and the protofilament closure into a cylinder. The predicted GPT-binding sites at the acidic tubulin C-terminus would be insufficient for such a recognition mechanism, and may require other components that associate with MTs, such as the N-terminal non-basic regions.

The efficient dissociation of EB1 from the MT lattice is partly caused by the negatively charged C-terminus of EB1. When this C-terminus was replaced with a neutrally charged motif, the EB1 mutant decorated the MT lattice without affecting its interaction with the growing MT ends [24]. The biochemical properties of GPT1/2 may render these molecules less able than EB1 to discriminate between MT ends and the lattice.

EB1 is not the only + TIP that autonomously interacts with the MT ends. *Xenopus laevis* XMAP215 (or ch-TOG in human) recognizes MT plus ends, but does not distinguish between polymerizing and depolymerizing ends [6]. XMAP215/ch-TOG binds to the extreme MT tip, whereas EB1 localizes to the tip region several tens of nanometers behind XMAP215/ch-TOG in vivo and in vitro [25, 26]. +TIPs that recognize distinct structural features at the plus ends would be expected to decorate different sub-regions at the MT ends.

### GPT does not require EB1 or SPR1 for tip tracking

EB1 recruits non-autonomous + TIPs via their C-terminal EB homology domain to growing MT ends [7]. CAP-Gly domains [27] and SxIP motifs [28] have been identified in EB1-interacting proteins that accumulate at growing MT ends. Interestingly, GPT1/2 still tracked the MT ends in the Arabidopsis *eb1* triple mutant background, which lacked EB1 function. The absence of CAP-Gly domains and SxIP motifs in GPTs suggests that their subcellular localization is independent of EB1. GPT1/2 localization to MT ends also does not require SPR1, another plant-specific + TIP [17, 18]. Although it is not known whether SPR1 autonomously tracks growing MT ends, these results suggest that if GPT does indeed use a hitchhiking mechanism to bind to a growing MT tip, it must couple with a + TIP protein other than EB1 or SPR1.

## Conclusions

Arabidopsis MAPs, GPT1 and GPT2, robustly track the plus end of growing MTs, independently of EB1 and SPR1, and thus define a novel plant-specific + TIP family.

## Methods

### Plant materials, growth conditions, and plant transformation

*Arabidopsis thaliana* ecotype Columbia was obtained from the ABRC stock center and used throughout the experiments. Arabidopsis seeds were sterilized with a solution of 0.1% Tween-20 and 10% sodium hypochlorite for 15 min, washed three times with sterilized water, and sown on Arabidopsis agar medium (2.5 mM KNO_3_, 1.25 mM KPO_4_, 1 mM Ca(NO_3_)_2_, 1 mM MgSO_4_, 35 μM Fe-EDTA, 7 μM MnCl_2_, 5 μM NaCl, 0.5 μM ZnSO_4_, 0.25 μM CuSO_4_, 0.1 μM NaMoO_4_, 0.005 μM CoCl_2_, 1.5% [*w*/*v*] agar, and 1% [*w*/*v*] sucrose). After stratification at 4 °C in darkness for 4 days, the seeds were germinated and grown vertically at 23 °C under a long photoperiod (16 h light/8 h darkness). Transgenic seedlings were identified by screening on ½ MS medium (Nihon Pharmaceutical Co.: 50 μg/mL myo-inositol, 0.2 μg/mL thiamine, 0.05% [*w/v*] MES-KOH pH5.7, 0.7% [*w/v*] agar, and 1% [*w/v*] sucrose) containing appropriate antibiotics. The seeds were germinated on the antibiotic-containing media and then scored after 14 days.

Arabidopsis was transformed using the floral dip method [29]. *Agrobacterium tumefaciens* GV3101 (pMP90) harboring the destination vector was selected on LB plates supplemented with the appropriate antibiotics. The bacteria were collected by centrifugation [29] and suspended in transformation solution containing 5% [*w/v*] sucrose and 0.05% [*v/v*] Silwet L-77. After the floral parts of the plants were dipped into the transformation solution, the plants were allowed to grow normally until the seeds were harvested.

For dual color visualization, a transgenic Arabidopsis plant expressing a GFP-tagged GPT protein was crossed with a plant harboring mCherry. To observe mitotic MT structures, mCherry-TUB6 was expressed under the control of the epidermis-specific promoter, a 3.4-kb 5′-upstream region from the putative transcription start site of *AtML1* [30]. To analyze cortical MT arrays in root epidermal cells, mCherry-TUB6 was expressed under the control of an epidermis-specific promoter (i.e., the 2.0-kb fragment immediately upstream of the translation start site of *WRKY72)* [31]. The GFP marker in the genomic *EB1b-GFP* construct [15] was removed by inverse PCR, followed by ligation of mCherry before the stop codon of EB1 to generate *EB1b-mCherry*. Cortical MT arrays in cotyledon epidermal cells were labeled with mCherry-TUB6 expressed under the control of the *UBIQUITIN 10* promoter [32].

The GPT-GFP constructs (described below) were directly transformed into the *eb1a-2 eb1b-3 eb1c-2* triple mutant, which was generated in our lab [15]. Transformants were first screened for hygromycin resistance, and then for GFP fluorescence. The *spr1-3* mutant, which had been screened in our lab [17], was transformed with pWRKY72::mCherry-TUB6, and subsequently crossed to the GPT-GFP expressing lines. Homozygous *spr1* mutant lines expressing mCherry-TUB6 and GPT-GFP were screened for the root skewing phenotypes and for simultaneous fluorescence of mCherry and GFP.

### Vector construction

For expression of GFP-tagged proteins in Arabidopsis plants, a full-length *GPT1* cDNA and a genomic fragment of *GPT2* that contained the 2.5 kb 5′-upstream region and the genomic region to just before the stop codon in the 8th exon, were cloned into pGWB5 and pGWB4 [33], respectively, using the Gateway Cloning System (Invitrogen). Transient expression vectors were constructed by fusing the appropriate cDNA fragment to GFP at the C-terminus of the expressed protein using the Gateway Cloning System in pUGW5 [33]. Truncated cDNA fragments were generated by PCR, and the ATG start codon was included before the N-terminus of a truncated cDNA.

### Recombinant protein purification and MT co-sedimentation assay

Full-length cDNAs of GPT1/2 were cloned into the pCold-MBP vector [34] using the appropriate restriction enzyme cleavage sites. The recombinant MBP-GPT proteins were expressed in the Rosetta (DE3) strain of *E. coli* and harvested and purified using an amylose resin column according to the manufacturer’s instructions (New England BioLabs). Purified fusion proteins were concentrated by centrifugal filters (Amicon Ultra filters; Merck Millipore), and the buffer was changed to BRB80 (80 mM PIPES, 1 mM MgCl_2_, 1 mM EGTA, pH 6.8). The final protein preparations were frozen in liquid nitrogen and stored at -80 °C.

For the MT co-sedimentation assay, thawed proteins were centrifuged at 100,000 x g for 15 min at 4 °C to remove protein aggregates. MTs were polymerized from bovine brain tubulin [35] in 1x BRB80 buffer supplemented with 1 mM GTP and 10 μM taxol (paclitaxel; Wako) at 37 °C for 35 min. Polymerized MTs were separated from unpolymerized tubulin dimers by centrifugation at 100,000 x g for 30 min at 30 °C. Precipitated MTs were suspended in the 1x BRB80 buffer supplemented with 10 μM taxol. MBP-GPT1 or MBP-GPT2 were mixed at 1 μM with MTs (equivalent to 1 μM tubulin) in 50 μL of 1x BRB80 buffer containing 10 μM taxol and incubated at room temperature for 1 h. MBP was purified as above and used as a negative control. After a 1-h incubation, the samples were centrifuged at 100,000 x g for 30 min at 30 °C. The pellet and supernatant fractions were mixed with SDS-PAGE sample buffer and analyzed by SDS-PAGE (using 10% polyacrylamide gels), followed by staining with Coomassie Brilliant Blue G-250 (Nacalai Tesque), as described previously [35]. Binding values were calculated from the equation *q* = (*q*_max_ x *c*)/(*K*_d_ + *c*) where *q* is the amount of protein bound to the tubulin dimer, *c* is the concentration of free MBP-GPT2 in solution, *K*_d_ is the dissociation constant, and *q*_max_ is the amount of bound MBP-GPT2 at the saturated level, by using a Prism 7 software (GraphPad Software Inc.).

### Particle bombardment-mediated transient expression

Plasmid (2 μg) harboring a full-length or truncated form of GPT1-GFP or GPT2-GFP and the tagRFP-MAP4 MT marker plasmid (2 μg) [21] were mixed for 30 min with 1.5 mg of 1.0 μm gold particles (Bio-Rad) that had been suspended in 19.2% glycerol, 962 mM CaCl_2_, and 1.5% spermidine, and were washed with 70% ethanol, followed by 100% ethanol. The plasmid-coated gold particles were bombarded into onion (*Allium cepa*) epidermal peels using the PDS-1000/He Biolistic Particle Delivery System (Bio-Rad) equipped with 1,100-p.s.i. rupture disks. After a 16-h dark incubation in a moist petri dish, the GFP-expressing cells were observed with a D-ECLIPSE C2 confocal microscope (Nikon). GFP was excited at 488 nm and observed using the ET514/30 band-pass emission filter, whereas tagRFP was excited at 543 nm and observed using the ET585/65 filter.

### Visualization of MTs using confocal spinning disc microscopy

Time-lapse imaging of fluorescent protein localization was performed on an inverted microscope (ECLIPSE Ti; Nikon) with a spinning disk confocal unit (CSU-XI; Yokogawa) connected to an EM-CCD camera (iXon3 DU897; Andor). Images were acquired using a numerical aperture oil-immersion objective (Apo TIRF 60x/1.49; Nikon). Excitation of fluorophores was accomplished using lasers (Andor) at 488 nm with a 520/35 filter for GFP or at 561 nm with a 617/73 filter for mCherry. Images were acquired at 2-s intervals for interphase cells or at 30-s intervals for mitotic cells. All images were collected by sequential acquisition using single channels. For dual-color visualization, mCherry was excited and its emission collected with an exposure time of 0.2 s. GFP was subsequently excited and its emission signals collected with an exposure time of 0.5 s. In another set of experiments, GFP was visualized first, followed by mCherry. The observation time lags for laser swapping caused temporal separation between the two fluorophores. Trajectories of individual MTs were traced on images and converted into kymographs using ImageJ. See Additional file 11 for schematic illustration of the procedures.

## Additional files

Additional file 1: Figure S1. Both N-terminal and C-terminal GFP fusions of GPT1 are localized to MTs in vivo. Full-length GPT1 protein was fused to GFP at its N-terminus (A) or C-terminus (B), and transiently expressed in onion epidermal cells. Scale bars, 50 μm. (PDF 103 kb)
Additional file 2: Figure S2. GPT2 tracks the growing plus ends of cortical MTs. (A) The subcellular localization of GPT2-GFP (green) and mCherry-TUB6, which labels cortical MTs (magenta), was analyzed in interphase cells of the Arabidopsis cotyledon epidermis. Scale bar, 5 μm. (B) Average fluorescence intensity (FI) profiles of GPT2-GFP and mCherry-TUB6 were obtained by analyzing and plotting data from 20 MT images. The data were normalized and the peak intensity of GPT2-GFP was set to 1. The error bars indicate SEM. (C) Representative time-lapse sequence and corresponding kymograph of the plus-end region of a growing MT. GPT2-GFP tracks the plus-end of a growing MT. The dashed yellow arrow indicates the position of the plus end. Scale bar, 2.5 μm. (PDF 173 kb)
Additional file 6: Figure S3. GPT2 does not require EB1 or SPR1 to track the MT end. (A and B) GPT2-GFP was expressed in the Arabidopsis *eb1a eb1b eb1c* triple mutant. (A) The subcellular localization of GFP-GPT1 was analyzed in interphase cells of the Arabidopsis cotyledon epidermis. Scale bar, 5 μm. (B) The average FI profile of GPT2-GFP was obtained by analyzing and plotting data from 20 MT images. The data were normalized and the peak intensity of GPT2-GFP was set to 1. The error bars indicate SEM. (C and D) GPT2-GFP was expressed in the Arabidopsis *spr1* mutant that also expressed the MT marker mCherry-TUB6. (C) The subcellular localization of GPT2-GFP and mCherry-TUB6 was analyzed in interphase cells of the Arabidopsis cotyledon epidermis. Scale bar, 5 μm. (D) Average FI profiles of GPT2-GFP and mCherry-TUB6 were obtained by analyzing and plotting data from 20 MT images. The data were normalized and the peak intensity of GPT2-GFP was set to 1. The error bars indicate SEM. (PDF 142 kb)
Additional file 11: Figure S4. Schematic illustration of the procedures generating merged intensity plots of two differently labeled proteins. (PDF 136 kb)

## Abbreviations

+TIPs: Plus-end tracking proteins
BPP: Basic proline-rich protein
EB1: End-binding protein 1
GFP: Green fluorescent protein
GPT1: Growing Plus-end Tracking 1
GPT2: Growing Plus-end Tracking 2
MAPs: Microtubule-associated proteins
MBP: Maltose-binding protein
MT: Microtubule
RFP: Red fluorescent protein
SPR1: SPIRAL1
TUB6: β-tubulin 6
