# Supplementary figures and images for: Novel Arabidopsis microtubule-associated proteins track growing microtubule plus ends
Source: BMC Plant Biol. 2017 Feb 2;17:33. doi: 10.1186/s12870-017-0987-5 (PMC5288973; doi:10.1186/s12870-017-0987-5)

# Additional file 1: Figure S1

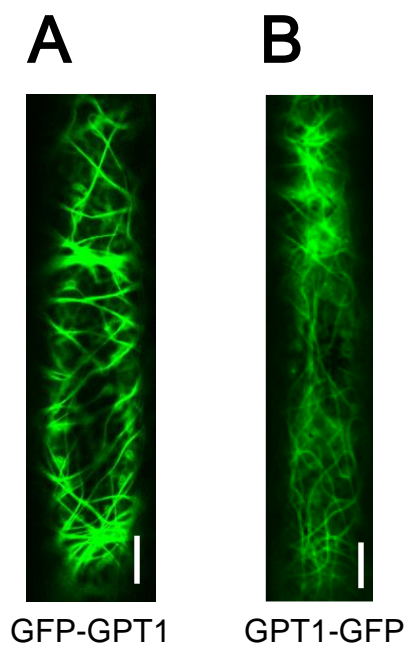

Supplement: Additional file 1: Figure S1. — Both N-terminal and C-terminal GFP fusions of GPT1 are localized to MTs in vivo. Full-length GPT1 protein was fused to GFP at its N-terminus (A) or C-terminus (B), and transiently expressed in onion epidermal cells. Scale bars, 50 μm. (PDF 103 kb) [file 12870_2017_987_MOESM1_ESM.pdf]

## Additional file 2: Figure S2

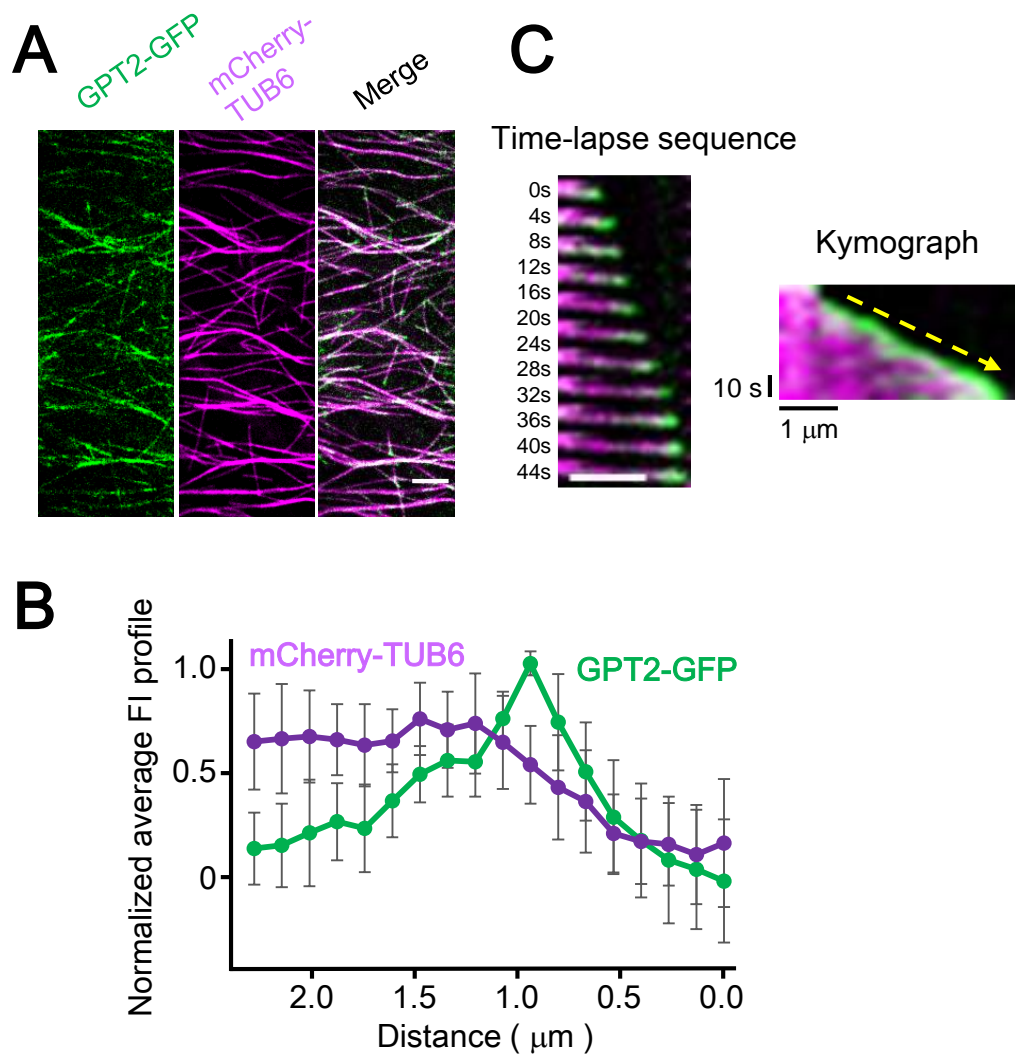

Supplement: Additional file 2: Figure S2. — GPT2 tracks the growing plus ends of cortical MTs. (A) The subcellular localization of GPT2-GFP (green) and mCherry-TUB6, which labels cortical MTs (magenta), was analyzed in interphase cells of the Arabidopsis cotyledon epidermis. Scale bar, 5 μm. (B) Average fluorescence intensity (FI) profiles of GPT2-GFP and mCherry-TUB6 were obtained by analyzing and plotting data from 20 MT images. The data were normalized and the peak intensity of GPT2-GFP was set to 1. The error bars indicate SEM. (C) Representative time-lapse sequence and corresponding kymograph of the plus-end region of a growing MT. GPT2-GFP tracks the plus-end of a growing MT. The dashed yellow arrow indicates the position of the plus end. Scale bar, 2.5 μm. (PDF 173 kb) [file 12870_2017_987_MOESM2_ESM.pdf]

# Additional file 3: Figure S3

**A**

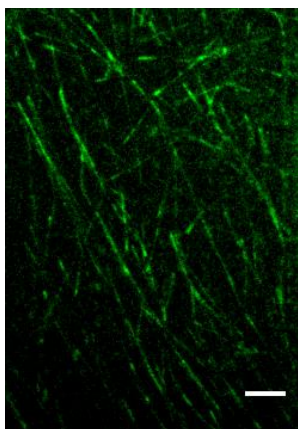

GPT2-GFP in  
*eb1a eb1b eb1c*

**B**

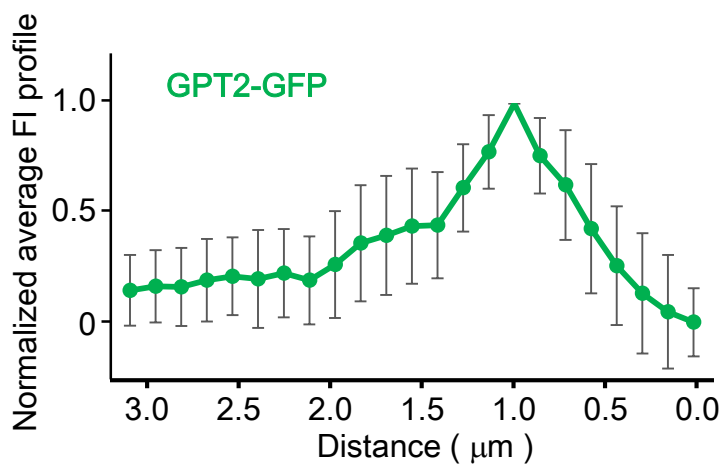

**C**

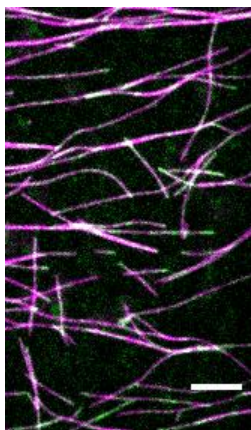

GPT2-GFP in *spr1*

**D**

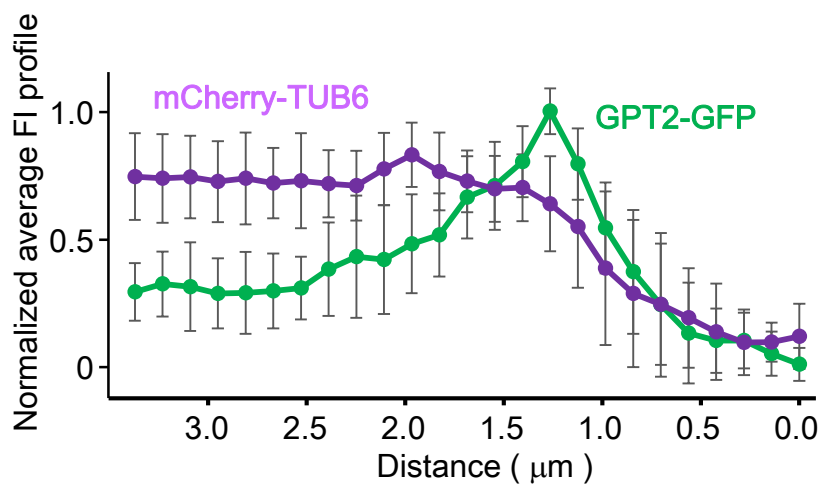

Supplement: Additional file 6: Figure S3. — GPT2 does not require EB1 or SPR1 to track the MT end. (A and B) GPT2-GFP was expressed in the Arabidopsis eb1a eb1b eb1c triple mutant. (A) The subcellular localization of GFP-GPT1 was analyzed in interphase cells of the Arabidopsis cotyledon epidermis. Scale bar, 5 μm. (B) The average FI profile of GPT2-GFP was obtained by analyzing and plotting data from 20 MT images. The data were normalized and the peak intensity of GPT2-GFP was set to 1. The error bars indicate SEM. (C and D) GPT2-GFP was expressed in the Arabidopsis spr1 mutant that also expressed the MT marker mCherry-TUB6. (C) The subcellular localization of GPT2-GFP and mCherry-TUB6 was analyzed in interphase cells of the Arabidopsis cotyledon epidermis. Scale bar, 5 μm. (D) Average FI profiles of GPT2-GFP and mCherry-TUB6 were obtained by analyzing and plotting data from 20 MT images. The data were normalized and the peak intensity of GPT2-GFP was set to 1. The error bars indicate SEM. (PDF 142 kb) [file 12870_2017_987_MOESM6_ESM.pdf]
