# Supplementary material for: Novel Arabidopsis microtubule-associated proteins track growing microtubule plus ends
Source: BMC Plant Biol. 2017 Feb 2;17:33. doi: 10.1186/s12870-017-0987-5 (PMC5288973; doi:10.1186/s12870-017-0987-5)
Supplement: Additional file 11: Figure S4. — Schematic illustration of the procedures generating merged intensity plots of two differently labeled proteins. (PDF 136 kb) [file 12870_2017_987_MOESM11_ESM.pdf]

Additional file 4: Figure S4

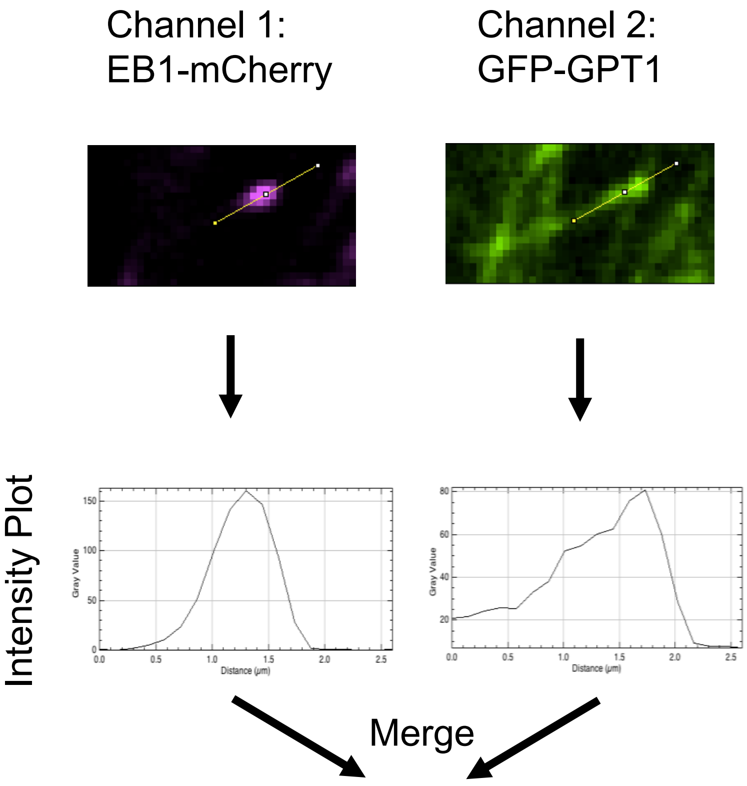

Line drawing at MT ends

- A straight line was drawn manually on Channel 1 first, then select Edit > Selection > Restore functions in ImageJ
- This will place the identical line on the same position on another channel

Intensity plot drawing

- Show relative fluorescent values along the line

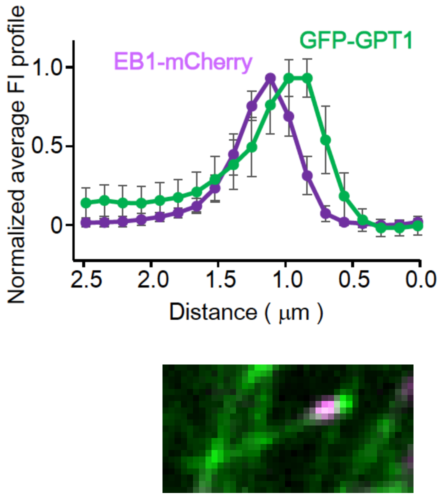

Merged intensity plots

- Retrieve intensity values at each position from both channels
- Values are normalized with the highest value set to 1.0
- Mean and STDEV values are calculated from 20 particles
